# The Effectiveness of Moxibustion: An Overview During 10 Years

**DOI:** 10.1093/ecam/nep163

**Published:** 2011-06-23

**Authors:** Song-Yi Kim, Younbyoung Chae, Seung Min Lee, Hyejung Lee, Hi-Joon Park

**Affiliations:** ^1^Department of Meridian and Acupoint, College of Korean Medicine, Kyung Hee University, 1 Hoegidong, Dongdaemungu, Seoul, 130-701, Republic of Korea; ^2^Acupuncture and Meridian Science Research Center (AMSRC), Kyung Hee University, 1 Hoegidong, Dongdaemungu, Seoul, 130-701, Republic of Korea

## Abstract

Moxibustion has been used to treat various types of disease. However, there is still insufficient evidence regarding its effectiveness. This study was performed to summarize and evaluate the effectiveness of moxibustion. A search was performed for all randomized controlled trials in PubMed between January 1998 and July 2008 with no language restriction. The results yielded 47 trials in which six moxibustion types were applied to 36 diseases ranging from breech presentation to digestive disorders. Moxibustion was compared to three types of control group: general care, Oriental medical therapies or waiting list. Moxibustion was superior to the control in 14 out of 54 control groups in 46 studies. There were no significant differences among groups in 7 studies, and the outcome direction was not determined in 33 studies. Seven studies were included in a meta-analysis. Moxibustion was more effective than medication in two ulcerative colitis studies (relative risk (95% CI), 2.20 (1.37, 3.52), *P* = .001, *I* ^2^ = 0%). Overall, our results did not support the effectiveness of moxibustion in specific diseases due to the limited number and low quality of the studies and inadequate use of controls. In order to provide appropriate evidence regarding the effectiveness of moxibustion, more rigorous clinical trials using appropriate controls are warranted.

## 1. Introduction

Acupuncture and moxibustion, representative therapeutic modalities in traditional medicine for more than 2500 years [1], are still being used in primary healthcare systems in East Asia [2]. Traditional treatments including acupuncture-related therapies (acupuncture, moxibustion and acupressure) and herbal remedies account for 40% of all healthcares in China [3]. In Korea, it was reported that 67% of Korean medical doctors use moxibustion as a therapeutic tool alone or in addition to their clinical practice [4, 5]. Acupuncture and moxibustion are similar regarding stimulation of acupuncture points on the Meridian [6]. Moxibustion uses thermal and chemical stimulants by burning herbal materials, including mugwort (*Artemisia vulgaris*, moxa), whereas acupuncture uses physical stimulation via insertion of needles [7]. The therapeutic components of moxibustion are assumed that the combination of heat (burning pain and heat stress), tar (extract), aroma (fume) and psychological stress [8]. Among them, heat stimulation and chemical action of ignited moxa is the most important variable for moxibustion [9]. As the method of acupuncture and moxibustion is different, there are functional distinctions between them. Acupuncture generally makes body release heat, or eliminates pathogen while moxibustion mainly makes body warm and recruits healthy qi [10]. Therefore, acupuncture and moxibustion are used to cover different conditions, while they share some common applications at the same time [11]. Recently, pre-clinical studies suggested that moxibustion boosts the immune system [8, 12] and enhances physiological functions [13]. In addition, accumulating clinical data support the use of moxibustion [14–19]. However, there have been few systematic reviews of the application of moxibustion, except in cases of breech presentation [20–22], and evidence regarding the efficacy of moxibustion is very limited.

The aims of this review are to summarize the present status of clinical research on moxibustion and to evaluate the evidence for the effectiveness of moxibustion.

## 2. Methods

### 2.1. Search Strategies and Selection of Studies

A search was performed in PubMed (http://www.ncbi.nlm.nih.gov/pubmed/), which offers better indexing and more search features [23], for the period spanning January 1998 to July 2008, with no language restriction. The literature search was performed using the key words “moxibustion” and “moxa”, and reference lists from the original articles and reviews were examined for additional studies.

Studies fulfilling the following criteria were included: (i) studies presenting the results of a randomized controlled trial (RCT); and (ii) studies using moxibustion in the intervention group. Studies in which moxibustion were administered to both the intervention and control groups were excluded. Studies using warm needles (i.e., examining not the efficacy of moxibustion itself, but its effects in addition to acupuncture) were also excluded. No limitations were applied to the type of disease, outcome measure or control group.

### 2.2. Data Extraction and Quality Assessment

The data were extracted systematically in a pre-defined, standardized manner according to the study designs, number of subjects, intervention, and control groups. Selection, extraction, and quality assessment were performed independently by two reviewers (Kim SY and Lee S). Discrepancies were resolved by discussion between reviewers. A modified Jadad scale was used to assess the methodological quality of the included studies [24, 25]. This scale assesses randomization (if the study was randomized, add 1 point; add an additional point for appropriate randomization and deduct 1 point for inappropriate randomization), patient and assessor blinding (add 1 point each), and reports of dropouts and withdrawals (add 1 point). The Jadad score ranges from 0 to 5 points. We considered trials with ≥3 points to be of high quality.

### 2.3. Data Analysis

As all available studies related to the efficacy of moxibustion were included, the diseases for this review were very heterogeneous. Therefore, we categorized each disease according to the International Statistical Classification of Diseases and Related Health Problems, 10th Revision (ICD-10). All the outcomes included were reviewed. To interpret the results efficiently and briefly, with compensation for heterogeneity, two alternative analyses were used: the relative risk (RR) and classification score. For studies providing effective rates as an outcome measure, the RR and 95% confidence interval (CI) were calculated, and re-analyzed using the *χ* ^2^ test. “Clinically cured” and “markedly effective” were included for success. When the RR could not be calculated, the appropriateness of outcomes related to the corresponding disease was discussed with experts from the Kyung Hee University Oriental Medical Hospital, followed by review of the statistical methods. For the classification score, the outcomes were defined as follows: (i) positive when moxibustion was significantly more effective than the control group (P); (ii) neutral when moxibustion was not significantly different from the control group (NEU); (iii) negative when the control group was significantly more effective than moxibustion (N); and (iv) not determined (ND) when the outcome measure was not appropriate for disease, the control was not adequate to prove the evidence of moxibustion, or the results were not clear. P and N were given only when the results of both author and reviewer were the same. If there was disagreement between authors and reviewers, the study was classified as ND. Meta-analyses were performed when the provided data were appropriate. The *I* ^2^ statistic describes the percentage of the total variability in study estimates that is due to heterogeneity rather than chance alone. The mean effect size was calculated using a random effects model, as we assumed that each study assessed different moxibustion treatments and thus different effects. Sensitivity analysis was performed using the *χ* ^2^ test to examine the statistical significance of associations between trial methodological quality, country, languages, type of intervention, comparative control group and trial outcome based on classification score. SPSS software (Version 13.0) and Review Manager (RevMan 5.0; The Nordic Cochrane Centre, Copenhagen) were used for statistical analyses, and *P* < .05 was taken to indicate significance.

## 3. Results

### 3.1. Study Characteristics

**Figure 1:**
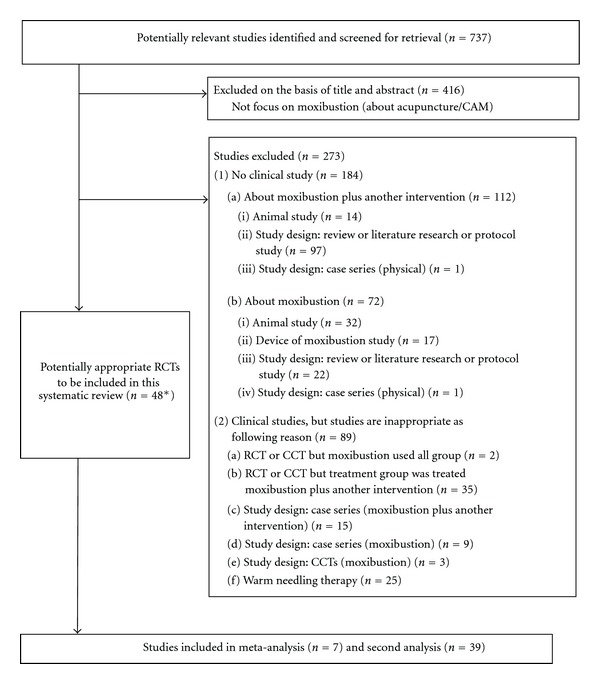
Flow diagram of literature search. *Two RCTs [27, 28] were considered as one study as they used the same population.

A total of 737 potentially relevant studies were identified and screened for retrieval. Forty eight of these studies [14, 16, 17, 26–70] fulfilled the inclusion criteria for this review (Figure 1) and are presented in Table 1. Two papers by Liu et al. [27, 28] on malignant tumors described the same populations and were considered as one study. Therefore, we included 47 studies in this review. In total, moxibustion was compared to 54 control groups in 47 studies where seven of these were three-armed studies [17, 31, 32, 34, 40, 49, 56].

Most of the studies were conducted in China (41 studies), three in Italy [14, 16, 17], and three in Korea [40, 61, 64]. Thirty-three papers were written in Chinese, and the rest were in English.

### 3.2. Participants and Conditions

A total of 4434 patients (2274 in the moxibustion group, 2160 in the control group) participated in the studies, and the data from 4360 patients (2239 in the moxibustion group, 2121 in the control group) were analyzed. The average number of subjects in each group ranged from 5 to 130 in the moxibustion groups (mean ± SD, 46.4 ± 28.6) and 5 to 130 in the controls (41.5 ± 25.6). The median sample sizes per group (moxibustion and control) were 38 and 34, respectively.

The ICD-10 was used to categorize disorders treated with moxibustion. Thirty-six types of disease were included within 13 subcategories of the ICD-10. The most frequently examined disorders were diseases of the musculoskeletal system and connective tissue (Chapter XIII, *n* = 7), the genitourinary system (Chapter XIV, *n* = 7), pregnancy, childbirth and the puerperium (Chapter XV, *n* = 7), and the digestive system (Chapter XI, *n* = 6). Others included diseases of the nervous system (Chapter VI, *n* = 4), the respiratory system (Chapter X, *n* = 4), endocrine, nutritional and metabolic diseases (Chapter IV, *n* = 3), neoplasms (Chapter II, *n* = 2), diseases of the circulatory system (Chapter IX, *n* = 2), the skin and subcutaneous tissues (Chapter XII, *n* = 2), certain infectious and parasitic diseases (Chapter I, *n* = 1), the blood and blood-forming organs and certain disorders involving the immune system (Chapter III, *n* = 1), and the eye, adnexa, ear and mastoid process (Chapters VII and VIII, *n* = 1). Among the 36 individual conditions included in these studies, the most frequently researched conditions were breech presentation (*n* = 7; [14, 16, 17, 67–70]), and tumors (*n* = 3; [27–29]).

### 3.3. Moxibustion Interventions and Control Groups

There are various methods of moxibustion treatment, including direct and indirect treatment with moxa cones, moxa sticks, moxa rolls with herbs, and natural moxibustion. We classified the treatments based on the World Health Organization (WHO) international standard terminologies on traditional medicine [6]. Direct and indirect moxibustion using a moxa cone was the most commonly used method in the studies included in this review. Direct moxibustion, in which an ignited moxa cone is applied directly to the skin surface at the acupuncture point [6], was used in seven studies [26, 39, 42, 48, 59, 61, 66]. In addition, indirect moxibustion, performed by placing some insulating material (ginger, salt, garlic, etc., according to symptoms) between the moxa cone and the skin [6], was used in 19 studies [29, 30, 32, 36, 40, 45, 47, 49–52, 54, 56, 58, 60, 62–65]. Moxa stick moxibustion involves the use of ignited moxa sticks named according to the type of stimulation method used (gentle, circling, pecking sparrow, and suspended moxibustion) [6] and was used in 19 studies [14, 16, 17, 27, 28, 31, 34, 35, 37, 41, 44, 46, 53, 55, 57, 67–70]. Moxa rolls with herbs (Taiyi moxa stick) were used in one study [33]. In addition, natural moxibustion, known as vesiculation moxibustion, in which irritants are applied at the acupuncture points to produce blistering and local congestion [6], was used in one study [43]. One study [38] employed multiple moxibustion techniques. The most frequently used acupuncture point was ST36 (*n* = 13), followed by CV8 (*n* = 11), BL23 (*n* = 10), BL20 (*n* = 8), CV4 (*n* = 7), GV14 (*n* = 7), BL67 (*n* = 7), SP6 (*n* = 5), BL13 (*n* = 4), and BL18 (*n* = 4). Participants received treatments 4 to 90 times (median, 20).

Three types of control group were included in the studies: (i) general care (*n* = 11), such as Western medication (*n* = 8), vitamin therapy (*n* = 1), injection of transfer factor (*n* = 1), and posture care (*n* = 1); (ii) oriental medical therapy (*n* = 20), such as acupuncture (*n* = 9), herbal medicine (*n* = 7), or combined traditional therapies (*n* = 4); and (iii) no treatment (*n* = 23), such as being placed on a waiting list (*n* = 2) or no additional treatment with the co-intervention (*n* = 21). There were no studies in which treatment effects were compared to a sham-control with indistinguishable appearance and no physiological effect.

### 3.4. Methodological Quality

Total 47 RCTs were included in this review. The scores for the methodological quality of the RCTs varied from 0 to 4. Most suffered from poor methodological quality. None of the RCTs were given the maximum of 5 points on the modified Jadad scale. Seven [16, 30, 35, 53, 54, 60, 61] of eight studies with more than 3 points for quality were published after 2005. Seven trials used a single blind method (patient or assessor), and there were no double (patient and assessor) blind studies included in the review. Only 31 RCTs described the method of randomization and nine used an inappropriate method, such as allocation according to treatment order. Power analysis was reported in only one study [16].

### 3.5. Outcomes

Eighty-three percent of the studies included in this review reported an effective rate (39 of 47 studies), and these were included in the secondary analysis comparing RRs among groups (Table 1). The classification score for the overall effects was decided considering both the authors' and the reviewers' interpretations. According to the classification score, moxibustion was superior to the control in 14 of 54 control group in 47 studies (26%). There were no statistically significant differences between groups in 7 studies (13%). The outcome direction was not determined in 33 studies (61%) for the following four reasons: (i) inappropriate outcome measures for the indicated disorder; (ii) details of the outcome measures were not described; (iii) the results were too complex to be determined; or (iv) inappropriate control to estimate the effectiveness. Among the 8 studies classified as high quality on the Jadad scale [14, 16, 30, 35, 53, 54, 60, 61], three studies could not be determined the direction of outcome, due to inappropriate control. Only five studies were estimated their classification score as two studies were positive, and three neutral. In details of high quality studies, indirect moxibustion for 20 days did not improve the symptoms of osteoarthritis compared to medication after treatment, but not in 2 months later, follow up point [54]. Patients for end stage renal failure in hemodialysis were measured by Kidney Disease Quality of life, but moxibustion didn't have additional effect with medication [60]. One study [61] for post-stroke urinary symptoms showed additional effect with oriental medical therapy as co-intervention, and this result was not contradictory to other low quality study [62]. Other two high qulity studies [14, 16] for breech presentation were attempted to polling, but not suitable as heterogeneity.

In detail, 10 days of direct moxibustion generated additional therapeutic effects to acupuncture in cases of herpes zoster [26] (RR (95% CIs), 1.67 (1.09–2.55), *P* = .016). Moxibustion showed no additional effects when used in conjunction with chemotherapy or radiotherapy in mid- to late-stage malignant tumors [27, 28] or nasopharyngeal carcinoma [29]. Biweekly indirect moxibustion for 2 months had an anti-aging effect compared to vitamin E (9.33 (1.33, 65.49), *P* = .002) [32]. For hyperlipidemia [33], Taiyi moxibustion for 3 months improved the patient response to diet therapy in terms of cholesterol, triglyceride, and high-density lipoprotein (HDL) levels. In Parkinson's disease, 30 courses of indirect moxibustion improved the effectiveness of medication based on the Unified Parkinson's Disease Rating Scale score (2.33 (1.04–5.21), *P* = .034) [36]. Stick moxibustion for 1 month did not improve the effects of a mixed therapy (electroacupuncture, medication, and vitamins) in facial paralysis [37], and 20 courses of stick moxibustion were not superior to medication in the treatment of diabetic peripheral neuropathy [34]. Direct moxibustion for 20 days provided additional improvement in the treatment of ischemic apoplexy, as determined by clinical symptoms and changes in transcranial Doppler (TCD) findings [39]. For allergic rhinitis and infantile repeated respiratory tract infection, moxa stick application for 10 days or 1 month was more effective than medication (2.09 (1.12–3.90), *P* = .025) [41] or intramuscular injection of transfer factor (1.85 (1.22–2.80), *P* = .002) [44]. Moxibustion for 6 days was superior to medication in infantile autumn diarrhea (2.25 (1.60–3.17), *P* < .001) [45]. In patients with chronic atrophic gastritis, moxibustion did not show an additional effect to acupuncture [46]. For ankylosing spondylitis [56], rheumatoid arthritis [57] and cervical spondylosis [59], moxibustion did not show additional benefits.

**Figure 2:**
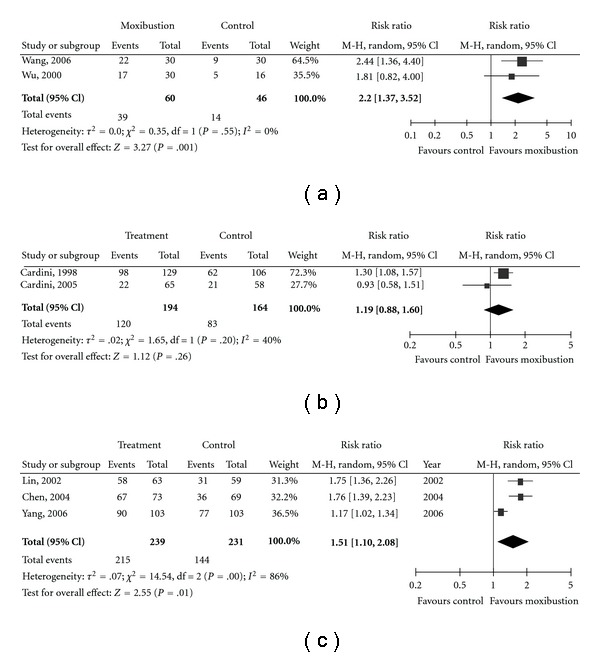
Forest plot of moxibustion compared to control. (a) Moxibustion compared with medication in ulcerative colitis [46, 49]. (b) Moxibustion compared with waiting-list control in breech presentation [14, 16]. (c) Moxibustion plus postual care compared with postual care only in breech presentation [68–70].

Only seven studies were performed for statistical pooling (Figure 2). Indirect moxibustion for 1 or 3 months was more effective than medication in two ulcerative colitis studies (2.20 (1.37, 3.52), *P* = .001, *I* ^2^ = 0%, *P* for heterogeneity = .55) (Figure 2(a); [47, 50]). Moxa stick treatment for 1–2 weeks did not show a effect on breech presentation compared to no treatment control (1.19 (0.88, 1.60), *P* = .26, *I* ^2^ = 40%, *P* for heterogeneity = .20) in two studies (Figure 2(b); 14,16). Moxa stick had additional effect to the posture care (1.51 (1.10, 2.08), *P* = .01), although marked heterogeneity was observed in this model (*I* ^2^ = 86%, *P* for heterogeneity = .0007, Figure 2(c); [68–70]).

The direction of study outcome (positive, neutral, not determined, or negative) was not significantly associated with intervention type (moxa cone versus moxa stick versus others, *χ* ^2^ = 1.835, df = 4, *P* = .895), country of origin (East Asia versus others, *χ* ^2^ = .572, df = 2, *P* = .758), or language (English versus other language, *χ* ^2^ = 2.573, df = 2, *P* = .392). In case of the association between direction of study outcome and study quality (high versus low, as assessed using the modified Jadad scale) seems to be near to *P* < .05 (*χ* ^2^ = 5.222, df = 2, *P* = .084), This may be caused by biased results that studies of low quality show a tendency of ND or positive. There was significant association between the direction of study outcome and control type (general care versus oriental medical therapy control versus no additional treatment with or without co-intervention, *χ* ^2^ = 21.209, df = 4, *P* < .001). It might be caused by inappropriate control, such as oriental medical therapy. Moxibustion studies compared to oriental medical therapy were assessed as ND. When sensitivity analysis were performed excluding “ND”, the direction of study outcome (positive, neutral, or negative) was not significantly associated with control group (general care versus no additional treatment with or without co-intervention, *χ* ^2^ = .936, df = 2, *P* = .713).

### 3.6. Adverse Effects

Only 12 studies [14, 16, 29, 30, 51, 52, 55, 57, 61, 62, 67, 68] commented on adverse events. Seven studies [14, 51, 52, 55, 61, 67, 68] reported no adverse event associated with moxibustion during the treatment period. Patients participating in a rheumatoid arthritis study [57] showed a slight and reversible increase in aminotransferase levels (3 of 30 cases) and a slight reduction in leukocyte count (two cases) after treatment with moxa sticks, whereas those in the control group (treatment with methotrexate and NSAIDs) had no appetite (nine cases), abnormal taste (nine cases), nausea (eight cases), increased aminotransferase levels (six cases) and thrombocytopenia (five cases). Liu et al. [62] reported slight burning and blistering in two patients treated with indirect moxibustion for post-stroke urinary tract symptoms. The two studies in which moxibustion was applied to nasopharyngeal carcinoma [29] and chemotherapy-induced leukopenia [30] also reported toxic side effects, but these were induced by radiotherapy or chemotherapy, rather than moxibustion. In the study on breech presentation, Cardini and weixin [14] reported no adverse event associated with moxibustion, and the numbers of cases of premature rupture of the membranes (PROM) and preterm delivery were less in the moxibustion group compared to the waiting list control (4 cases versus 12 cases of PROM and two cases versus three cases of preterm delivery among 130 subjects, resp.) [14]. However, in another study performed by the same group [16], two cases of preterm delivery possibly due to PROM and one case of bleeding were noted in association with moxibustion, whereas one case of preterm delivery occurred in the waiting list control group. In this study, moxibustion was associated with complaints of unpleasantness and physical disturbances due to the odor (42%), throat problems (22%), and abdominal pain because of contractions (17%). Twenty-two percent of participants temporarily or definitively interrupted the treatment because of these symptoms. Peng's study [67] for breech presentation reported five cases of abdominal pain in control group.

## 4. Discussion

Although many studies have provided encouraging results regarding the use of moxibustion in various disorders, definitive conclusions cannot be drawn from the evidence presented in this review. The use of moxibustion has been studied in a wide range of diseases, from neoplasm to pain, but we cannot properly evaluate the effectiveness of moxibustion in each disease due to the limited number of corresponding studies. In addition, the overall quality of these studies was low, and the use of controls was inappropriate. Nevertheless, meta-analysis suggested beneficial effect that moxibustion appeared to be more effective than medication in ulcerative colitis. In breech presentation, there is strong heterogeneity exist, thus the results are inconclusive. There have been three relevant systematic reviews on this topic, however, the results are not directly comparable because two were not focused on the moxibustion as an intervention [21, 22], and one missed the relevant studies included in our review [20].

Among the 36 individual conditions included in these studies, the most frequently studied were breech presentation (*n* = 7; [14, 16, 17, 67–70]), and tumors (*n* = 3; 27–29). As only one or two studies covered each disease, definitive conclusions cannot be drawn.

We found that the quality of studies regarding moxibustion was unsatisfactory. Thirty-nine of 47 studies (83%) received scores of less than 3 on a modified Jadad scale. In most studies, the details of the moxibustion procedure used were not fully described. Methods regarding randomization were unclear and power analysis was seldom reported. Concealment of allocation and blinding methods were not clearly described, and details regarding drop-out and withdrawal rates were often insufficient. Even the trials that scored as high quality on the modified Jadad scale were not devoid of flaws; for example, none of the studies attempted patient blinding and only one study reported power analysis; several studies that had been expressed as positive in their original papers were re-classified as ND because the outcome measures were not appropriate either for the disease or the results were contradictory; seventy-four percent of studies did not report adverse events related to intervention. Despite these shortcomings, we found that methodological quality has improved recently, and seven of the eight high-quality studies were published after 2005.

All of the studies used inappropriate controls, which makes evaluation difficult because controls in RCTs provide important insight into the effectiveness of the treatment and help to eliminate factors that may otherwise skew the results [71]. Furthermore, it is extremely difficult to compare each other and estimate their effectiveness because of the inappropriateness in the quality and control. In our analysis, 20 studies, which had been compared to oriental medical therapy, were re-classified as ND because the controls were not appropriate to estimate effectiveness. The issue of appropriate control methods for moxibustion has not drawn a great deal of attention. Since many components, such as heat, fumes, and moxa extract [8], may contribute to the therapeutic effect, developing appropriate controls for moxibustion is not easy tasks. Two types of sham moxibustion designed to isolate heat [72, 73] have been introduced. However, it is not obvious whether subjects were really blinded because the thermal intensity was different. Therefore, there is a need for a more appropriate sham moxibustion method for use as a control.

Among the 12 RCTs that reported adverse events, none reported serious adverse events. There were complaints related to unpleasantness and physical disturbances due to the odor [16]. Blistering and slight burning were also reported in one studies [62]. Because moxibustion involves thermal stimulation of the skin over a prolonged period, it is important that only experienced and well-trained practitioners provide this therapy [62]. In addition, for widespread adoption of moxibustion, it will be necessary to develop an odor-free device for its application.

This review had several limitations. Although we made efforts to retrieve all relevant RCTs, some studies in other databases may have been missed. However, it is reported that Pubmed offers better indexing and more search features, and there is overall a relatively high degree of overlap between Medline and EMBASE and CENTRAL [23, 74]. Further limitations include the paucity and the overall insufficient methodological quality of the primary data. As the quality of clinical trials and reporting methodologies included in this review was generally weak, further high-quality trials are needed to assess the effectiveness of moxibustion in treating several diseases. In this regard, future trials should adhere to rigorous trial designs that are suitable for the research questions. To improve the research quality, future researchers should follow the guidelines for reporting clinical trials, such as the Consolidated Standards of Reporting Trials (CONSORT) statement. We suggest that specific guidelines for the reporting of moxibustion trials, similar to the Reporting Interventions in Controlled Trials of Acupuncture (STRICTA) [75], should be developed and followed by researchers.

In conclusion, even though our results for the effectiveness of moxibustion are not conclusive due to the heterogeneity and the poor quality of the studies, we intend that this review gives researchers and clinicians more information of the real clinical usages of moxibustion, and thus it could be extended over a wide field of disorder for practice and research. Of course, more specific and rigorous trials with large sample sizes are needed to evaluate the effectiveness of moxibustion for each disease.

## Funding

This research was supported by Basic Science Research Program through the National Research Foundation (NRF) funded by the Ministry of Education, Science and Technology (R11-2005-014).

## Figures and Tables

**Table 1:** Characteristics and findings of included studies (*n* = 47).

Author, year, Coun	Disorders (Quality*)	Intervention, *N* (analyzed), Freq, Dur	Control, *N* (analyzed)	Co-intervention	Author's findings		Reviewer's analysis	
					Outcomes	Results	Effective rate (mox versus con, *n*/*N*)**, *P* value***, RR [95% CI]	Classification score
I-B. Certain infectious and parasitic diseases								

Zhang [26] 2007, Cn	Herpes zoster (2)	Mox (Cd), 38, 1/day, 10 days	NT, 34	Acu	(1) Effective rate (symptom) (2) Effect-producing time (pain and symptom, resp.)	(1) *P* < .05 (2) Each *P* < .05	28/38 versus 15/34,*P* = .016 1.67[1.09, 2.55]	P

II-C. Neoplasms								

Liu [27, 28], 2001, 2002, Cn	Mid-late stage malignant tumor (2)	Mox (S), 30(20), NA	NT, 35(27)	Chemo + Herb^a^	(1) Therapeutic effect (2) No. of leucocyte and lymphocyte (3) T-lymphocyte subset (4) Normalized cases of plasma fibrinogen (5) Karnofsky score (6) Quality of life	(1, 2, 3) NA (4, 5) *P* < .05 (6) NS except pain (*P* < .05)	4/30 versus 5/35,*P* = 1.000 0.93[0.28, 3.16]	NEU
Chen [29] 2000, Cn	Nasopharyngeal carcinoma (NPC) (2)	Mox (Ci), 28, 1/day, 1mon	NT, 28	Radio	(1) Remission rate (tumor size) (2) Toxic and side effects occurrence (3) 5-year local control rate of NPC and cervical lymph node (4) 5-year survival and metastasis rate (5) MDA, MMS, -SH and SOD activity in blood	(1, 3, 4) NS (2) *P* < .05 (5) MMS, -SH:*P* < .05, MDA, SOD: *P* < .01	23/28 versus 21/28,*P* = .746 1.10 [0.83, 1.44]	NEU

III-D. Diseases of the blood and blood-forming organs and certain disorders involving the immune mechanism								

Zhao [30] 2007, Cn	Leukopenia induced by chemotherapy (4)	Mox (Ci), 114(113), 1/day, 10 days	Herb, 114 (108)		(1) Cured rate and effective rate (No. of leucocyte) (2) Physical strength (zubrod-ECOG-WHO) and the gastrointestinal toxic effects according to WHO grading criteia (nausea, vomiting, diarrhea and constipation) (3) Side effects: functions of the heart and lung and kidney induced chemotherapy (the toxic effects according to WHO grading criteria)	(1) Each *P* < .01 (2) *P* < .05 (3) NS	95/113 versus 38/108, *P* = .000 2.39 [1.83, 3.12]	ND

IV-E. Endocrine, nutritional and metabolic diseases								

Liao [31] 2007, Cn	Diabetes (2)	A: Mox (S), 24 B: Mox (S), 26, 2/day, 10×3 days	C: Acu, 29 C: NT, 29	Acu	(1) Effective rate (2) Cured and markedly effective rate, symptom, FBG, 24 hour urinal glucose, glycosylated hemoglobin, TC, TG, HDL and LDL	(1) NS (2) A versus C (NS) B versus C (*P* < .01)	A versus C: 7/24 versus 9/29,*P* = 1.000 0.94 [0.41, 2.15] B versus C: 12/26 versus 9/29,*P* = .279 1.49 [0.75, 2.94]	NDND
Gao [32] 2007a, Cn	Aging (0)	Mox (Ci), 30, 2/wk, 2 mon	A: Acu, 21 B: Vit E, 20		(1) Effective rate (symptom) (2) Symptomatic score (type of kidney deficiency) (3) Serum SOD activities and MDA contents	(1) *P* < .05 (versus A), *P* < .01 (versus B) (2) *P* < .01 (versus A, B) (3) *P* < .05 (versus A, B)	A: 14/30 versus 3/21, *P* = .019 3.27 [1.07, 9.96] B: 14/30 versus 1/20,*P* = .002 9.33 [1.33, 65.49]	NDP
Li [33] 2005a, Cn	Hyperlipidemia (1)	Mox (St), 80, 1/day, 30 × 3 days	NT, 80	Diet	(1) Hypercholesterolemia subgroup (each *n* = 20): TC (2) Combined hyperlipidemia subgroup (each *n* = 20): TC (3) Hypertriglyceremia subgroup (each *n* = 20): TG (4) Hypo-HDL cholesterolemia subgroup (each *n* = 20): HDL	Each *P* < .05	NA	P

VI-G. Diseases of the nervous system								

Zhao [34] 2008, Cn	Diabetic peripheral neuropathy (2)	Mox (S), 20, 1/2 days, 10 × 2 times	A: Acu, 20 B: Med, 20		(1) Symptom score and total effective rate (symptom) (2) FBG, HbA1c (3) Hemorheological indexes (a) Whole blood viscosity (b) Capillary blood viscosity (c) Hematocrit (4) ET, NO, MDA	(1, 2) NS (versus A), *P* < .05 (versus B) (3), (1) NS (versus A), *P* < .05 (versus B) (2), (3) NS (versus A, B) (4) each NS (versus A) and *P* < .05, 0.05, NS (versus B)	A: 10/20 versus 9/20,*P* = 1.000 1.11 [0.58, 2.14] B: 10/20 versus 7/20,*P* = .523 1.43 [0.68, 3.00]	NDND
Tian [35] 2006, Cn	Herpes simplex virus facial neuritis (4)	Mox (S-tube), 80(78), 1/day, 6 × 8 times	Mox (S), 80(79)	Acu	(1) Total effective rate (symptom, HBS and FDI) (2) HBS (3) FDI	(1, 2) *P* < .05 (3) *P* < .05 (FDIs), NS (FDIp)	51/78 versus 35/79,*P* = .010 1.48 [1.10, 1.98]	ND
Zhang [36] 2005, Cn	Parkinson's disease (0)	Mox (Ci), 54, 1/2 days, 15 × 2 times	NT, 36	Med	(1) Total effective rate (UPDRS) (2) The modified UPDRS score	(1, 2) *P* < .01	21/54 versus 6/36,*P* = .034 2.33 [1.04, 5.21]	P
Gao [37] 2005, Cn	Facial paralysis (0)	Mox (S), 47, 1/day, 30 days	NT, 40	EA + Med^c^ + Vit B	Total effective rate (symptom)	*P* < .05	38/47 versus 25/40,*P* = .091 1.29 [0.98, 1.71]	ND

VII, VIII-H. Diseases of the eye, adnexa, ear and mastoid process								

Zhang [38] 2008, Cn	Vertigo (2)	Mox (TF, Cd, Ci), 66, 4 × 3 days	Mox (Cd), 51		(1) Total effective rate (symptom) (2) Total effective rate according to pattern identification (3) Vertigo symptom rating score	(1, 3) *P* < .05 (2) NA	30/66 versus 17/51,*P* = .254 1.36 [0.85, 2.18]	ND

IX-I. Diseases of the circulatory system								

Chen [39] 2006, Cn	Ischemic apoplexy (0)	Mox (Cd), 14, 1/day, 20 days	NT, 14	Routine care	(1) Change of cerebrovascular functions by TCD (2) Clinical effect by nervous function detect	(1, 2) *P* < .05	NA	ND
Moon [40] 2003, Kr	Stroke (1)	Mox (Ci), 10, 1/2 days, 15 days	A: EA, 15 B: NT, 10	Acu	(1) Change of MAS according to treatment period (2) Correlation between MAS and treatment period	(1) NA (2) NA	NA	NDND

X-J. Diseases of the respiratory system								

Yang, [41] 2008, Cn	Perennial allergic rhinitis (2)	Mox (S), 60, 1/day, 10 days	Med^d^, 60		(1) Total effective rate after treatment and 3 mon later (symptom) (2) Clinical symptoms (tickle of nose, sneezing, snivel, congestion)	(1) *P* < .05 and *P* < .01 (2) each *P* < .05	23/60 versus 11/60,*P* = .025 2.09[1.12,3.90]	P
Li [42] 2005b, Cn	Asthma of lung deficiency type (2)	Mox (Cd), 37, 1–2/day, 7 × 3 days	Acu, 35		(1) Total effective rate (symptom) (2) Clinical symptoms (3) Pulmonary function (FVC, FEV_1_ and FEF_0.25–0.75_) (4) Each content of Ig and C3	(1) NS (2, 3, 4) each NA	18/37 versus 11/35,*P* = .156 1.55[0.86,2.79]	ND
Lai [43] 2001, Cn	Allergic asthma (1)	Mox (N), 121, 1/wk, 4 wk	Drug acupoint application, 88		(1) Total effective rate after treatment and 6 mon (symptom) (2) Total effective rate according to pattern identification (a) Lung heat and kidney yin deficiency (b) Lung cold, lung qi deficiency and kidney yang deficiency	(1) Co > Ex:*P* < .05 and NS (2) (a) *P* < .01(Co > Ex) (b) NS	After treatment 52/121 versus 51/88,*P* = .036 0.74 [0.57, 0.97] After 6 mon 38/95 versus 32/74,*P* = .753 0.93 [0.65, 1.32]	ND
Long [44] 2001, Cn	Infantile repeated respiratory tract infection (1)	Mox (S), 46, 5/wk, 1 mon	Transfer factor, 40		(1) Effective rate (symptom) (2) Each level of IgG, IgA and IgM (*n* of each group = 24)	(1) *P* < .05 (2) *P* < .01	34/46 versus 16/40,*P* = .002 1.85 [1.22, 2.80]	P

XI-K. Diseases of the digestive system								

Chui [45] 2008, Cn	Infantile autumn diarrhea (2)	Mox (Ci), 683 × 2 days	Med^e^, 68		(1) Total effective rate and effective rate (symptom) (2) The mean of symptoms-stopping time (3) Negative conversion rate of HRV Antigen after 72 hr	(1) each *P* < .05 (2) *P* < .05 (fever and diarrhea), NS (nausea) (3) *P* < .01	54/68 versus 24/68,*P* = .000 2.25 [1.60, 3.17]	P
Gao [46] 2007b, Cn	Chronic atrophic gastritis (1)	Mox (S), 30 2 mon	NT, 30	Acu	(1) Total effective rate (symptom) (2) Pathological effect (3) Level of serum gastrin	(1, 3) *P* < .05 (2) NA	13/30 versus 15/30,*P* = .796 0.87 [0.50, 1.49]	ND
Wang [47] 2006, Cn	Ulcerative colitis (0)	Mox (Ci), 30 1/day, 10 × 3 days	Med^f^, 30		(1) Effective rate and total effective rate (symptom) (2) Ig G, Ig A and Ig M content (3) Peripheral blood T-cell and NK cell	(1) *P* < .05 and .01 (2) each *P* < .05, NS and NS (3) NA	22/30 versus 9/30,*P* = .002 2.44 [1.36, 4.40]	P
Yu [48] 2002, Cn	Diarrhea (1)	Mox (Cd), 56 1/2 days, 1 mon	Herb, 56		(1) Total effective rate (symptom) (2) Salivary amylase (3) Middle molecular substances in serum	(1, 3) *P* < .05 (2) *P* < .01	32/56 versus 25/56,*P* = .257 1.28 [0.88, 1.85]	ND
Lin [49] 2000, Cn	Postoperative gastric functional disorder (1)	Mox (Ci), 20 7 days	A: Herb, 20 B: Med^g^, 20		Change of preoperative and postoperative EGG	NS	NA	NDNEU
Wu [50] 2000, Cn	Ulcerative colitis (1)	Mox (Ci), 30 1/day, 12 × 5 days	Med^h^, 16		(1) Cure rate (symptom and colomoscopy result) (2) Change of colonic mucosal histopathology and mucin (*n* = 13)	(1) *P* < .01 (2) NA	17/30 versus 5/16,*P* = .1291.81 [0.82, 4.00]	ND

XII-L. Diseases of the skin and subcutaneous tissue								

Diao [51] 2007, Cn	Neurodermatitis (2)	Mox (Ci), 30 1/2 days, 1 mon	Med^i^, 30		(1) Total effective rate (symptom) after 2 wk, 3 wk and final treatment (2) Relapse rate during treatment period	(1) each *P* < .01,*P* < .05 and *P* < .05 (2) *P* < .05	27/30 versus 20/30,*P* = .057 1.35 [1.02, 1.79]	ND
Lao [52] 2005, Cn	Chloasma (2),	Mox (Ci), 60 2/wk, 8 × 3 times	NT, 46	Acu	Total effective rate (symptom)	*P* < .05	31/60 versus 21/46,*P* = .562 1.13 [0.76, 1.69]	ND

XIII-M. Diseases of the musculoskeletal system and connective tissue								

Chen [53] 2008, Cn	Myofascial pain syndrome (3)	Mox (S), 57 1/day, 10 times	Acu + Cupping + TDP, 50		(1) PRI, VAS and PPI of McGill pain questionnaire (2) Cured or markedly effective rate and total effective rate (symptom and PRI, VAS, PPI of McGill pain questionnaire)	(1) Each *P* < .001 (2) *P* < .001 and NS	49/57 versus 12/50,*P* = .000 3.58 [2.16, 5.93]	ND
Sun, [54] 2008a, Cn	Knee osteoarthritis (3)	Mox (Ci), NA(29) 1/day, 10 × 2 times	Med^j^, NA(27)		(1) Improvement in the symptom after 5, 10, 20 times and 2 mon later (2) Cured or markedly effective rate (after treatment and 2 mon later) (symptom)	(1) *P* < .05, NS, NS and *P* < .05 (2) NS and *P* < .05	After treatment (no. of knees) 26/41 versus 19/39,*P* = .260 1.30 [0.88, 1.94] After 2 mon later 23/41 versus 13/39, *P* = .047 1.68 [1.00, 2.83]	NEU
Li, [55] 2008, Cn	Knee osteoarthritis (1)	Mox (S), 31(30) 6/wk, 4 wk	Herb^k^, 31(30)		(1) Cured rate and effective rate (symptom) (2) VAS, ISOA scale and effect-producing time	(1) *P* < .05 and NS (2) each *P* < .05	27/30 versus 18/30,*P* = .015 1.50 [1.09, 2.06]	ND
Jia [56] 2006, Cn	Ankylosing spondylitis (1)	Mox(Ci), 30 1/2 days, 2 × 3 mon	A: Acu, 30 B: NT, 30	MTX + Med^l^	(1) Total effect rate (symptom, ESR and CRP) (2) Cured or markedly effective rate (3) The days needed for reaching the clinical remission and remarked effect (4) Relapse rate after 1 year (5) Improvement in the symptoms and signs (a) Morning stiffness, swollen and painful peripheral joints and chest expansion (b) Finger-ground distance, sacroiliitis index, schober test and occipital wall test (6) Change in ESR and CRP	(1) NS (versus A), *P* < .05 (versus B) (2,3) NA (versus A), *P* < .05 (versus B) (4) each 0, 0 and 30 (5) (a) NS (versus B) (b) *P* < .05 (versus B) (6) NA	A: 25/30 versus 24/30, *P* = 1.000 1.04 [0.82, 1.32] B: 25/30 versus 18/30, *P* = .084 1.39 [1.00, 1.94]	NDND
Li [57] 2006a, Cn	Rheumatoid arthritis (2)	Mox (S), 30 5/wk, 3 mon	NT, 30	Med^m^	(1) Improved rate, markedly effective rate, effective rate, ACR 50% effective rate and the ratio withdrawing NSAIDs (2) Symptoms (a) Swelling joint and grasping power (b) Body pain, tenderness of joint and lasting time of the grasping power (3) HAQ (4) ESR, CRP and RF (5) Rate of adverse reaction	(1) NS, NS, *P* < .05, *P* < .05 and *P* < .01 (2) (a) *P* < .01 (b) NS (3) NS (4) *P* < .05, NS and NS (5) *P* < .05	22/30 versus 14/30,*P* = .0641.57 [1.01, 2.44]	ND
Zeng [58] 2006, Cn	Cervical vertigo (1)	Mox (Ci), 40 10 × 2 days	Acu, 38		Cured rate and total effective rate (symptom, blood supply and recurrence after 3 mon)	Each *P* < .05	39/40 versus 28/38,*P* = .003 1.32 [1.09, 1.61]	ND
Zhuang[59] 2000, Cn	Cervical spondylosis (1)	Mox (Cd), 21 10 times	NT, 19	Acu	Cured or markedly effective rate	*P* < .01	18/21 versus 11/19,*P* = .078 1.48 [0.97, 2.26]	ND

XIV-N. Diseases of the genitourinary system								

Sun [60] 2008b, Cn	End stage renal failure in hemodialysis (3)	Mox (Ci), 37 2–3/wk, 4 × 3 wk	NT, 34	Hemodialysis + Med	Kidney Disease Quality of life Short Form (KDQOL)	*P* < .05: only 3 items (Dialysis staff encouragement, Role-Emotional and General health) among the 20 items	NA	NEU
Yun [61] 2007, Kr	Post-stroke urinary symptoms (4)	Mox (Cd), 21(20) 5/10 days	NT, 20(19)	Herb + Acu	(1) IPSS (frequency, weak stream, quality life scale, obstructive score, irritative score and total IPSS score) (2) QOL index (3) Urinary symptoms improvement according to the severity (4) Barthel Index	(1) *P* < .05 (2) NS (3) *P* < .05 (mild, moderate), NS (severe) (4) NS	NA	P
Liu [62] 2006, Cn	Post-stroke urinary symptoms (2)	Mox (Ci), 41(32), 5/wk, 3 wk	NT, 41(30)	Acu	(1) The therapeutic effects (mean of urinary times every day and mean times to be asked to awaken for the nursing personnel at night) (2) Mean times of urgent urinary incontinence at day for the patient and cases-times of urinary incontinence of the patient at night (3) Increasing degree of urinary incontinence	(1) Each *P* < .01 (2, 3) NA	NA	P
Li [63] 2006b, Cn	Primary dysmenorrhea (2)	Mox (Ci), 78, 7(max)/cycle, 3 mon	Herb^n^, 60		(1) Cured rate and total effective rate (2) Index of therapeutic effect	(1, 2) Each *P* < .05	71/78 versus 45/60,*P* = .018 1.21 [1.03, 1.43]	ND
Shin [64] 2006, Kr	Pain and coldness on hysterectomy (2)	Mox (Ci), 5, 1/day, 5wk	Acu, 5		(1) Change of body temperature (2) Change in pain	(1) NS (2) NA	NA	ND
Wang [65] 2005, Cn	Primary dysmenorrhea (1)	Mox (Ci), 48, 1/day, 10 × 3 times	Herb^n^, 48		(1) Cured or markedly effective rate and effective rate (2) Blood PGF_2a_ (3) OT of serum	(1) *P* < .01 and*P* < .05 (2) NA (3) *P* < .05	42/48 versus 14/48,*P* = .000 3.00 [1.91, 4.72]	ND
Zhao [66] 2004, Cn	Impotence (1)	Mox(Cd), 51, 1/day, 10 × 3 times	Herb, 31	Vit B_12_	(1) Cured rate (2) Total effective rate	(1) NA (84% versus 71%) (2) NA (96% versus 90%)	43/51 versus 22/31,*P* = .169 1.19 [0.92, 1.53]	ND

XV-O. Pregnancy, childbirth and the puerperium								

Neri [17] 2007, It	Breech presentation (2)	A: Mox (S), 16(15), 2/wk B: Mox (S), 15(14), 2/wk	C: Acu, 10 C: NT	Acu	(1) Fetal heart rate (2) Fetal movement (3) Success rate (cephalic presentation)	(1, 2) NA (3) NA (80% versus 57% versus 28%)	A versus C: 12/15 versus 2/10,*P* = .005 4.00 [1.13, 14.17] B versus C: 8/14 versus 2/10,*P* = .104 2.86 [0.76, 10.70]	NDND
Peng [67] 2006, Cn	Breech presentation (1)	Mox (S), 40, 2/day, 7 days	K-C position, 40		Success rate (cephalic presentation)	NS	16/40 versus 20/40,*P* = .500 0.80 [0.49, 1.31]	NEU
Yang [68] 2006, Cn	Breech presentation (2)	Mox (S), 103, 2/day, 7 days	NT, 103	K-C position	Success rate (cephalic presentation)	*P* < .05	90/103 versus 77/103,*P* = .032 1.17 [1.02, 1.34]	P
Cardini[16] 2005, It	Breech presentation (4)	Mox (S), 65, 1-2 wk	NT, 58		(1) RR (cephalic presentation in the 35th wk) (2) Cephalic presentations at delivery (3) AFM count during the first week of each group (4) Compliance with treatment and adverse events	(1) NS (34% versus 36%, RR 0.95, 99% CI [0.59–1.5]) (2) NA (52% versus 51%) (3) NS (254 versus 220, *P* = .04) (4) NA	22/65 versus 21/58,*P* = .851 0.93 [0.58, 1.51]	NEU
Chen [69] 2004, Cn	Breech presentation (0)	Mox (S), 73, 2/day, 3 days	NT, 69	R position	(1) Success rate (cephalic presentation) (2) Treatment period for co-intervention	(1, 2) *P* < .01	67/73 versus 36/69,*P* = .000 1.76 [1.39, 2.23]	P
Lin [70] 2002, Cn	Breech presentation (0)	Mox (S), 63, 2/day	NT, 59	K-C position	(1) Success rate (cephalic presentation) (2) The mean of treatment period	(1, 2) *P* < .01	58/63 versus 31/59,*P* = .000 1.75 [1.36, 2.26]	P
Cardini [14] 1998, It	Breech presentation (3)	Mox (S), 130, 1-2/day, 1-2 wk	NT, 130		(1) Cephalic presentation at 35 wk, birth and at birth (exclude subjects treated with external cephalic version, *n* = 129, 106, each group) (2) AFM during a 1 hr for 1 wk (3) Compliance with treatment and side effects (4) Number and causes of caesarean deliveries and use of oxytocin for vaginal deliveries (5) Apgar score <7 at 5 min	(1) Each *P* < .001, = .020 and .004 (2) *P* < .001 (3) NA (4) NS and *P* < .001 (5) *P* = .006	98/129 versus 62/106,*P* = .005 1.30 [1.08, 1.57]	P

ACR, American College of Rheumatology; Acu, acupuncture; AFM count, the fetal motor activity; AIDS, acquired immuno-deficiency syndrome; Auth, author; CCT, clinical controlled trial; CD4, cluster of differentiation 4; Chemo, chemo therapy; Cn, China; Co, control group; Coun, Country; CRP, C-reactive protein; C3, serum complement-3; Diet, diet therapy; Dur, Duration; EA, electroacupuncture; EGG, electrogastrography; ESR, erythrocyte sedimentation rate; ET, plasma endothelin; Ex, experiment group; FBG, fasting blood-glucose; HbA1c, hemoglobin A1c; FDI, Facial disability index; FEF_25–75%_, maximal midexpiratory flow rate; FEV_1_, forced expiratory volume in 1 second, Freq, Frequency; FVC, forced vital capacity; HAART, Highly Active Antiretroviral Therapy; HAQ, Health Assessment Questionnaire; Herb, herb medicine; HBS, House-Brackmann scale; HDL, high density lipoprotein; hr, hour (s); HRV, human rotavirus; Ig, serum immunoglobulin; IPSS, International Prostate Symptom Score; ISOA scale, index of severity of osteoarthritis; It, Italy; KDQOL-SF, Jp, Japan; KDQOL, Kidney Disease Quality of Life Short Form; K-C position, Knee-chest position; Kr, Korea; LDL, low density lipoprotein; MAS, modified Ashworth scale; Med, medication; MDA, malondialdehyde; MMS, middle molecular substance; mon, month (s); MTX, Methotrexate; NA, not available; NK cell, natural killer cell; NO, nitric oxide; NRS, numeric rating scale; NS, not significant; NSAIDs, Non-steroidal anti inflammatory drugs; NT, no additional treatment; OT, oxitocin; PPI, present pain intensity; PRI, pain rating index; QOL, quality of life; Radio, radio therapy; RF, rheumatoid factor; R position, Raising buttocks method; RR, relative risk; -SH, sulfhydryl; SOD, superoxide dismutase; TC, total cholesterol; TCD, transcranial Doppler ultrasound; TDP, Teding Diancibo Pu; TG, triglyceride; UPDRS, United Parkinson's Disease Rating Scale; VAS, visual analogue scale; vit, vitamin; Waiting, waiting list control; wk (s), week (s); WHO, Would Health Organization.

Abbreviation list of moxibustion type: Mox (Cd), moxa cone moxibustion (direct); Mox (Ci), moxa cone moxibustion (indirect); Mox (S), moxa stick moxibustion; Mox (ST), Taiyi moxa stick; Mox (S-tube), moxa stick of tube type; Mox (TF), thunder-fire wonder moxibustion; Mox (N), natural moxibustion.

Abbreviation list of control medication: a, Guben Yiliu III (GBYL), a Chinese herbal composite preparation in combination with chemotherapy; b, Mecobalamin tablets; c, Dexamethasone or Prednisone; d, Cetirizine hydrochloride; e, Smecta; f, Sulphasalazine and Metronidazole tablets; g, Motilium; h, Sulphasalazine; i, Triamcinolone and urea cream; j, Sodium Diclofenate slow-released tablet; k, Xianling Gubao capsules; l, Sulphasalazine; m, Methotrexate (MTX) and non-steroid anti-inflammatory agents (NSAIDs) n, Yueyueshu powder.

*Quality was assessed using a modified Jadad scale; **Number of high responders/number of total analyzed participants; ***P-value was calculated by *χ* ^2^ test.
